# Visual-SLAM Classical Framework and Key Techniques: A Review

**DOI:** 10.3390/s22124582

**Published:** 2022-06-17

**Authors:** Guanwei Jia, Xiaoying Li, Dongming Zhang, Weiqing Xu, Haojie Lv, Yan Shi, Maolin Cai

**Affiliations:** 1 School of Physics and Electronics, Henan University, Kaifeng 475004, China; jiaguanwei@henu.edu.cn (G.J.); lixiaoying1@stu.scu.edu.cn (X.L.); haojielv@henu.edu.cn (H.L.); 2 School of Automation Science and Electrical Engineering, Beihang University, Beijing 100191, China; shiyan@buaa.edu.cn (Y.S.); caimaolin@buaa.edu.cn (M.C.); 3 Pneumatic and Thermodynamic Energy Storage and Supply Beijing Key Laboratory, Beijing 100191, China

**Keywords:** visual-SLAM, classical framework, key techniques, developmental needs

## Abstract

With the significant increase in demand for artificial intelligence, environmental map reconstruction has become a research hotspot for obstacle avoidance navigation, unmanned operations, and virtual reality. The quality of the map plays a vital role in positioning, path planning, and obstacle avoidance. This review starts with the development of SLAM (Simultaneous Localization and Mapping) and proceeds to a review of V-SLAM (Visual-SLAM) from its proposal to the present, with a summary of its historical milestones. In this context, the five parts of the classic V-SLAM framework—visual sensor, visual odometer, backend optimization, loop detection, and mapping—are explained separately. Meanwhile, the details of the latest methods are shown; VI-SLAM (Visual inertial SLAM) is reviewed and extended. The four critical techniques of V-SLAM and its technical difficulties are summarized as feature detection and matching, selection of keyframes, uncertainty technology, and expression of maps. Finally, the development direction and needs of the V-SLAM field are proposed.

## 1. Introduction

**Figure 1:**
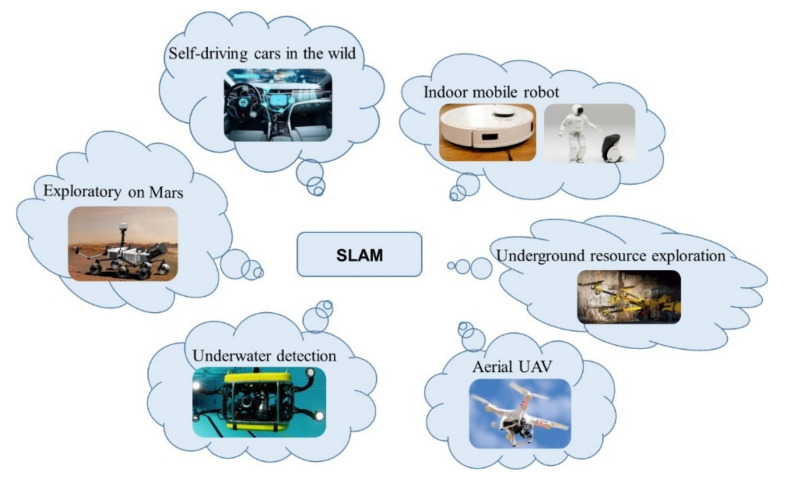
Multiple applications of SLAM.

Advances in computer technology have expanded the field of “unmanned operation,” in which machines replace humans, so that humans can significantly broaden the scope of their work. Most machines currently complete tasks based on available scene maps, but robots built for work in unknown environments have higher requirements, because it is difficult to achieve full automation. SLAM (Simultaneous Localization and Mapping) is a technology that can achieve the autonomous positioning of mobile machines [1] for navigation, path planning, and target tracking. V-SLAM can use a visual sensor that works as a human eye to obtain information about the robot’s environment and, then, build a model, while accurately estimating its movement [2], all without any prior information about the environment. It can also move into to unknown settings that humans cannot reach; so, it has been studied in-depth. V-SLAM is widely used on the ground [3,4], under the water [5,6], or in the air [7,8], as shown in Figure 1. It has broad applications in resource detection, obstacle avoidance navigation, and uncrewed operations.

This review begins with the development of SLAM, a review of V-SLAM from its proposal to the present day, and a summary of its historical milestones. Then, it explains the five parts of the classic V-SLAM framework: the frontend (including visual sensor and visual odometer), backend optimization, loop detection, and mapping. The four critical techniques of V-SLAM and their technical difficulties are summarized in context. Finally, the development direction and needs of V-SLAM research are proposed.

## 2. The Development of V-SLAM

In 1986, Smith [9], who was studying spatial uncertainty’s description and transformation representation, published a groundbreaking article on SLAM and proposed the concept of a probabilistic SLAM. In 1988, he [10] proposed to use it to estimate a state vector. Then Durrant-Whyte [11,12] proposed a filter-based backend optimization algorithm, and his research results prompted Smith [9] to write papers on landmarks. The similarity in their errors in estimating the robot’s position proved that the milestones must be correlated. However, the amount of calculation to achieve the state vector was huge. Researchers decoupled the landmarks, so that mapping and positioning were treated as independent parts, and SLAM development entered a bottleneck period. A breakthrough came when researchers realized that the error was a matter of estimation, and the SLAM problem was seen as convergent, a theory first proposed by Csorba [13].

In 2006, V-SLAM [14] was proposed as a branch of research, and it attracted the attention of researchers, who published numerous papers. A variety of SLAM algorithms and solutions based on visual sensors were also proposed, such as Mono-SLAM [15] (Monocular-SLAM), based on monocular cameras, and PTAM [16] (Parallel Tracking and Mapping), which introduced the possibility of running various SLAM tasks in parallel. The solution used Bundle Adjustment (BA) [17] based on nonlinear optimized keyframes to solve position and map structures. ORB-SLAM [18] (Oriented FAST and Rotated BRIEF) was proposed based on PTAM, which is currently the most effective feature-based method. In addition, V-SLAM using the direct method has also been suggested––LSD-SLAM [19] (Large-Scale Direct Monocular SLAM) is one of these. Based on this, DSO-SLAM (Direct Sparse Odometry SLAM) [20] has also been proposed and is currently the best solution for estimating accuracy and operating efficiency. The V-SLAM system can run on various equipment terminals [21,22]. Kimera has been designed and is suitable for the broader SLAM field with metric semantics modularity [23].

**Figure 2:**
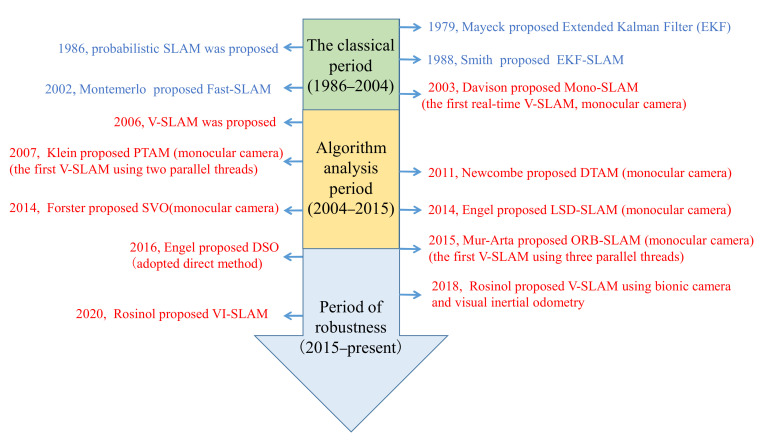
Development of V-SLAM.

More and more university laboratories are engaged in V-SLAM research, for example, the Dyson Robotics Laboratory of Imperial College, London, UK; the Automation System Laboratory, ETH Zurich, Switzerland; the Machine Vision Research Group of the Technical University of Munich, Germany; and the Laboratory for Information & Decision Systems (LIDS), Massachusetts Institute of Technology, Cambridge, MA, USA [24]. The overall development process of V-SLAM is shown in Figure 2.

## 3. V-SLAM Classical Framework

**Figure 3:**
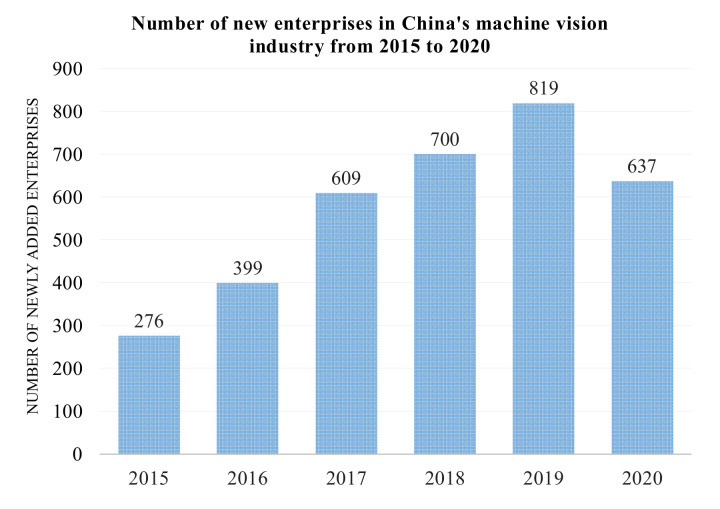
The number of newly added enterprises in China’s machine vision industry.

V-SLAM is currently one of the critical technologies in robotics, autonomous driving, and augmented reality and is the basis for intelligent mobile platforms to perceive the surrounding environment. Since images or videos can provide rich environmental information, most research on positioning and mapping focuses on the V-SLAM algorithm. With the increase in the popularity and applicability of machine vision, the number of enterprises engaged in this research is also increasing, for example, in China. In 2020, although affected by the COVID-19 pandemic, 637 new companies were started, as shown in Figure 3.

**Figure 4:**
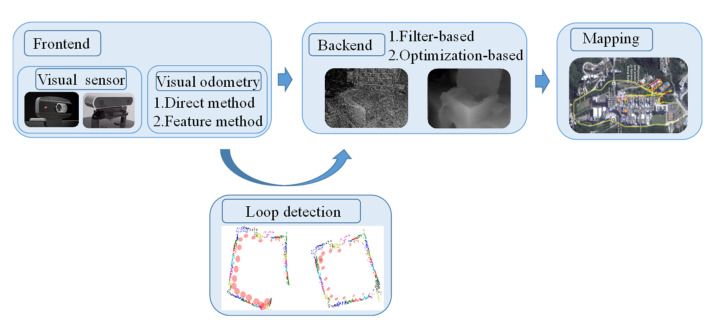
Framework of V-SLAM.

The development of machine vision has accelerated the research of V-SLAM and its classical framework, as shown in Figure 4. The procedure of V-SLAM is generally divided into two parts: the frontend and the backend. At the frontend, the visual sensor is mainly responsible for collecting data during movement and transmitting the data to the visual odometer, which estimates the data of adjacent images or points to form a local map and assess the robot’s position. The backend is responsible for optimizing the frontend information and, finally, produces a complete map. The purpose of loop detection is to judge whether the positions the robot has walked are coincident by comparing before and after information to avoid drift.

### 3.1. Frontend

The frontend of V-SLAM is to process the input image and obtain the motion relationship while the camera moves to determine the position of the current frame. It mainly contains two parts, namely a visual sensor and a visual odometer. The visual sensor is responsible for reading and preprocessing the camera image information. The visual odometer [25] estimates the camera movement based on the data from adjacent images to provide a better initial value for the backend.

#### 3.1.1. Visual Sensor

With advances in computer technology, visual sensors have significantly improved in resolution, pixels, and focus. According to different working methods, these are divided into monocular, stereo, and RGB-D (Red Green Blue-Depth) cameras [26]. The monocular camera has a simple structure and fast calculation speed but lacks the depth of information and has scale blur [27]. Stereo cameras can obtain depth information indoors or outdoors through the four steps of calibration, correction, matching, and calculation, but the amount of computation needed is significant. RGB-D cameras have become popular in the last ten years [28,29] because they can obtain image color and depth information at the same time [30]. V-SLAM, based on an RGB-D camera, has developed rapidly [31,32,33] because of two key technologies: structured light and time of flight (TOF). However, it is susceptible to light interference and a limited measurement range. The depth detection range of Kinect, which uses RGB-D cameras as visual sensors, is only 1.2–3.5 m, and the visible spectrum is only 43° in the vertical and 57° in the horizontal direction [34].

#### 3.1.2. Visual Odometry

After the visual sensor collects the image information, the visual odometer [25] determines the position and direction of the robot by analyzing the camera image. It pays attention only to the local consistency of the trajectory, and the operational model reconstructs the path incrementally. According to the type of feature extraction needed, it is classified into the feature-point or direct method. The first method [25] extracts sparse features from an image, completes frame-matching through the descriptor, and then calculates the position according to the constraint relationship among the elements. The ORB-SLAM (Oriented FAST and Rotated BRIEF) proposed by Mur-Artal [18] is a well-known system that uses the feature point method and is the core feature of the V-SLAM. The direct method introduces the idea of optical-flow tracking. Based on the assumption of constant luminosity, the optimization goal is to minimize the luminosity error to solve position variables. DSO (Direct Sparse Odometry) [20] is one of the few systems that use the pure direct method to calculate visual odometry. SVO (Semi-direct Visual Odometry) [35] uses the direct method in the Sparse model-based Image Alignment part of the frontend.

The frontend using the feature-point method is the mainstream one of the visual odometer. Specific feature detection and matching are summarized in Section 3.1. Compared with the complete V-SLAM, VO has a better real-time performance. If research is only required on the camera path instead of the environment map, VO is the better choice. It sacrifices global consistency to obtain real-time computing performance.

Visual inertial SLAM (VI-SLAM) is favored in research and application [36] to better solve the obstacles of pose accuracy and adaptability in complex dynamic scenes [37]. The highlight is the complementary function between V-SLAM and IMU (Inertial Measurement Units) [38]. The SLAM is used to help the IMU eliminate accumulated errors and complete closed-loop detection; the IMU is used to support the SLAM in solving the positioning accuracy with less texture and fast movement [39]. Jun et al. improved the initial convergence speed for a higher positioning accuracy with a monocular camera and IMU [40]. Chai et al. employed enhanced vanishing point optimization to correct the cumulative drift error of the VI- SLAM system effectively [41] Xu et al. achieved an improved positioning accuracy of 10% in VI-SLAM with a fusion of the feature point and optical flow method [42]. Eckenhoff et al. performed real-time high-precision positioning and efficient result calibration using a multi-camera visual–inertial navigation system [43]. Zhu et al. achieved better accuracy and robustness by combining a stereo camera and IMU components with sparse mapping in the VI-SLAM algorithm [44].

Multisensor fusion based on VI-SLAM has become a research hotspot for achieving higher positioning accuracy and robustness. Shan et al. enhanced the extraction of visual depth information. They improved the accuracy of visual recognition by using the close coupling of the visual–inertial system and the inertial LIDAR system [45]. Zhang et al. integrated sensor fusion with a laser rangefinder and monocular camera SLAM to solve the limited camera depth range [46]. Yang et al. achieved accurate and robust localization results for complex indoor scenes by fusing super-bandwidth with VI-SLAM [47]. With the deepening of research and application, the fusion of VI-SLAM with a more complex structure and high-precision multisensors will continue to be a research direction.

At the frontend, the working principles of monocular, stereo, and RGB-D cameras are introduced in the visual sensor part. The feature-point method and the direct method are used to reconstruct the path by VO. It is also one of the decisive factors for the development of V-SLAM.

### 3.2. Backend Optimization

The backend receives the original data collected by the visual sensor provided by the frontend and performs the calculation and optimization. In V-SLAM, the frontend and computer-vision research fields are more related. At the same time, the backend optimization is essentially a state estimation problem that uses either the filter-based or the nonlinear optimization method.

Earlier on, the filter-based method was the main one in the backend of V-SLAM. It is summarized in papers [11,12]. Its core purpose was iterating and updating the state quantity continuously, as uniformly described by the Bayesian filter model. The filter-based method represented by KF (Kalman Filter) [48] can optimize and process the frontend data. However, errors occur, and the real-time performance of the algorithm cannot be guaranteed. To solve the above problems, the Extended Kalman Filter (EKF) proposed by Moutarlier [49] is of great help in dealing with uncertain information.

**Figure 5:**
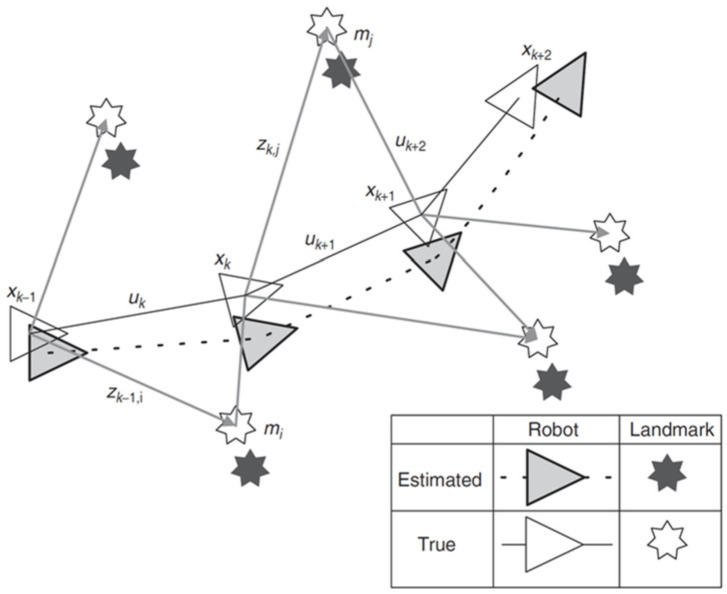
Essential issues of SLAM.

For EKF, a mobile robot moves in the environment and uses visual sensors on the robot to observe known landmarks (as shown in Figure 5). At time *k*, the state vector *x_k_* describes the position and direction of the vehicle, and the control vector is *u_k_*. The position vector is an *m_i_* of the *i*th landmark. The part of the *i*th landmark is *z_ik_*. Gray and dark gray are the predicted positions of the mobile robots and landmarks, while white represents the actual positions of the robots and landmarks.

Equation (1) indicates that the basis of the EKF SLAM is to describe the vehicle movement:

(1) $$p(x_{k}|x_{k-1},u_{k})\Leftrightarrow x_{k}=f(x_{k-1},u_{k})+w_{k}.$$

The *f*(*·*) function is the kinematics model of the vehicle, *w_k_* stands for the additivity, and the observation model is as described in Equation (2):

(2) $$p(z_{k}|x_{k},m)\Leftrightarrow z_{k}=h(x_{k},m)+v_{k}$$

**Figure 6:**
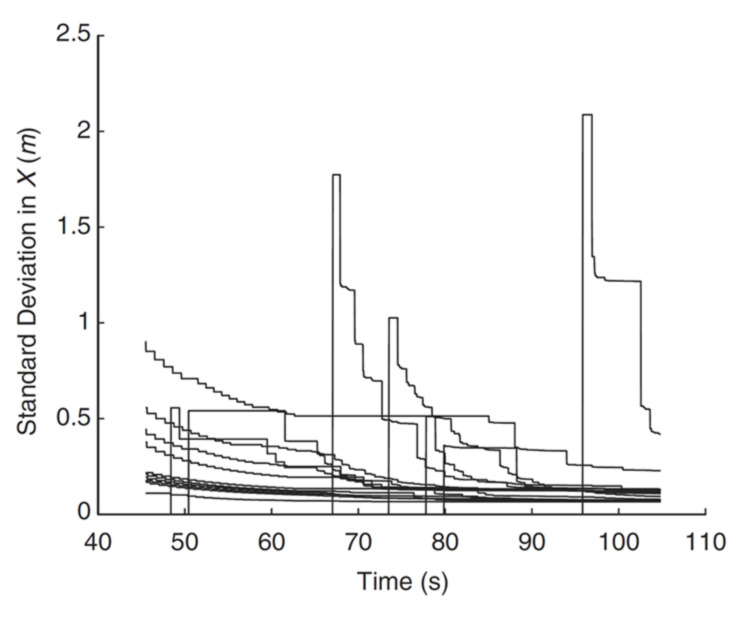
The standard deviation of the landmark location changes with time.

The *h*(*·*) function is the observed geometric shape, and *v_k_* represents the additivity. Figure 6 shows that a series of the standard deviation of landmark locations varies over time.

**Figure 7:**
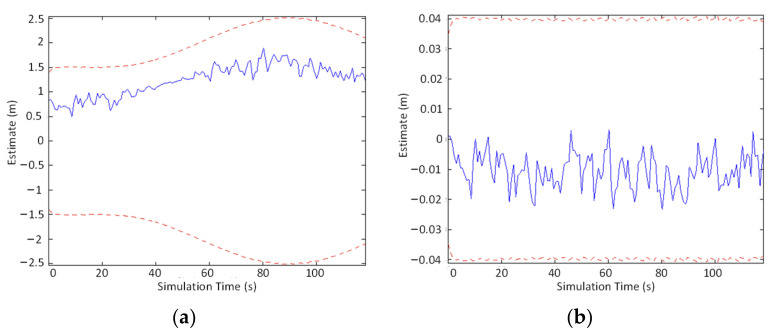
Results for the first 600 s (3000 timesteps): (**a**) error in *x*_v_, (**b**) error in *θ*_v_.

**Figure 8:**
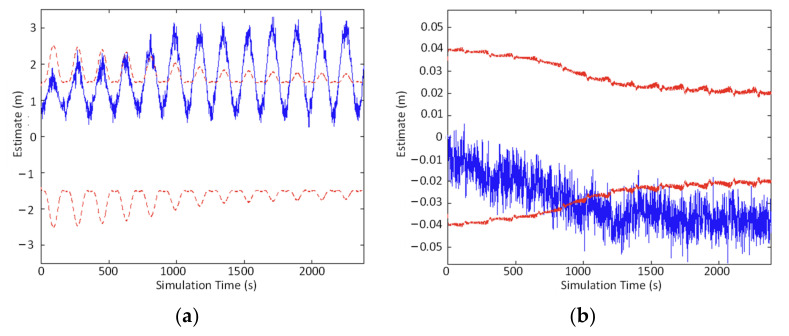
Results for the first 3200 s (16,000 timesteps): (**a**) error in *x*_v_, (**b**) error in *θ*_v_.

For V-SLAM, the number of map points and positions will increase while the system is running. The covariance scale and the mean that the EKF needs to maintain and update will also become more extensive. Meanwhile, the linear approximation between the motion and observation works in a small range, and severe nonlinear errors will be caused at longer distances. In paper [50], the experimental data of backend optimization using EKF showed that when the timesteps were 3000, the estimated error of the vehicle position *x_v_* and orientation *θ_v_* was within the standard deviation limit during the first 600 s of vehicle operation (Figure 7). However, when the timesteps were 16,000, the estimated errors of the vehicle position *x_v_* and orientation *θ_v_* were not within the standard deviation limit in the first 3200 s (Figure 8).

Due to the limitations of the EKF algorithm for nonlinear, non-Gaussian systems, researchers also proposed the PF (Particle Filter) [51] method. Particles were used to describe the position or map points, and the probability density distribution of the approximate state was solved by randomly sampling the particles. The advantage of the PF method is that state estimation is not sensitive to data association but has better performance in linear approximation.

Considering the different characteristics and advantages of the EKF and PF algorithms, Montemerlo [52,53] applied an RBPF [54] (Rao-Blackwell’s Particle Filter) to a robot SLAM and named it “the Fast-SLAM algorithm”. The algorithm broke the SLAM problem into a robot localization and an environmental feature position estimation problem. The PF algorithm was used to estimate the position of the entire path, combining the advantages of EKF and probabilistic methods. It not only reduced the computational complexity but also had better robustness.

**Figure 9:**
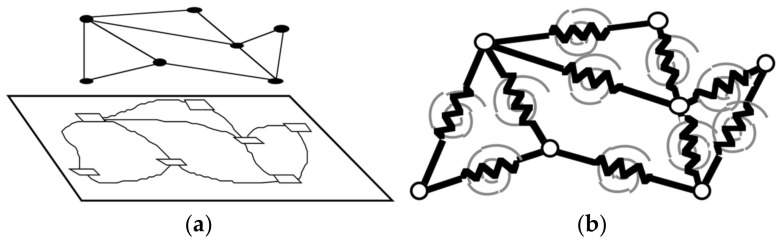
Spring–node model: (**a**) Graph-based representation of an environment and (**b**) equivalent truss.

With the development of digital image processing, filter-based technology is gradually being replaced by graph-based optimization [55]. In 1998, Golfarelli [56] proposed a truss model with each route as an adjustable bar and each landmark a node. A spring connected two adjacent nodes that showed the constrained relationship between them. The spring stiffness coefficient represented the uncertainty of the constraint. The springs and nodes represent routes and landmarks, respectively, as shown in Figure 9.

**Figure 10:**
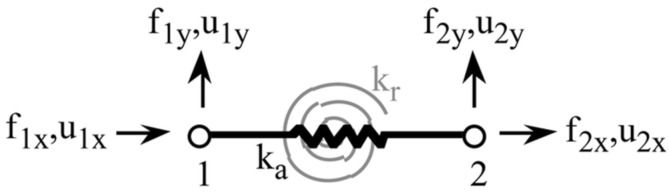
The truss has a linear elastic spring (black) and a rotational elastic spring (gray).

From a mechanical point of view, the model is constructed by combining an axial linear spring with a rotary spring (Figure 10). *k_a_* and *k_r_* are the spring constants. The model includes a linear spring (black) and a rotating spring (gray).

Then, there is Equation (3):

(3) $$k_{a}\propto \frac{1}{\Delta x},k_{r}\propto \frac{s^{2}}{\Delta y},$$

where

(4) $$\Delta x=\int_{-\infty }^{+\infty }\int_{-\infty }^{+\infty }\delta (x,y,C)\left|x\right|dxdy$$

(5) $$\Delta y=\int_{-\infty }^{+\infty }\int_{-\infty }^{+\infty }\delta (x,y,C)\left|y\right|dxdy.$$

**Figure 11:**
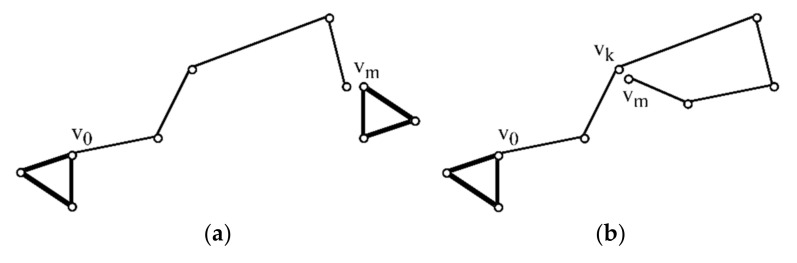
Error correction of the open polygon (**a**) and closed polygon (**b**).

*C* is the covariance matrix of the route *r*. Suppose the robot started to explore the unknown area from the known landmark *v*_0_ and finally met landmark *v_m_*. If *v_m_* had been reached before, the orderly connected line segments between *v*_0_ and *v_m_* would form an open polygon (Figure 11a). If not, a closed polygon is created (Figure 11b).

When reflecting the correction effect, there are two critical parameters: the average percentage error *σ* on this path and the average percentage error *ρ* in the direction of the path. The changes in the parameters before and after modification are shown in Table 1.

**Figure 12:**
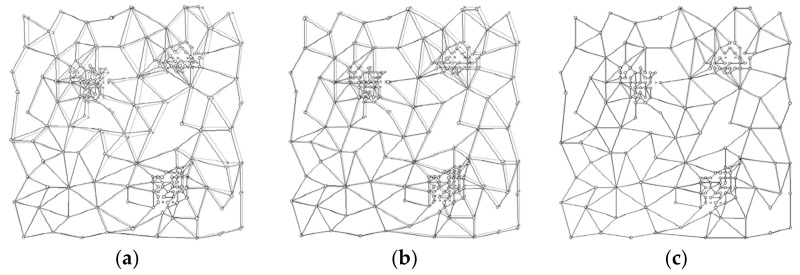
Comparison of a 1-time correction (**a**), 5-times correction (**b**) and 50-times correction (**c**).

Figure 12 shows the correction comparisons performed 1, 5, and 50 times. The actual map is gray, and the corrected one is black.

**Figure 13:**
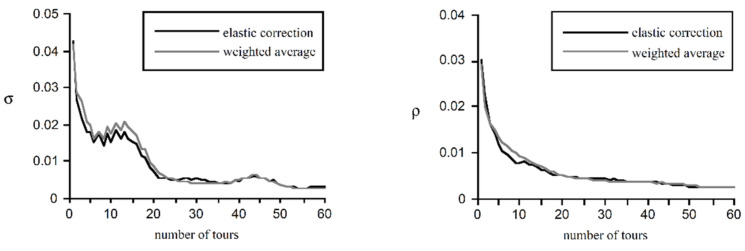
Comparison of elastic correction and weighted average.

As shown in Figure 13, in the first ten tours, the average error of the landmark positions measured before the correction was reduced to 20–30%, and in the subsequent tours, it dropped below 10%.

The backend optimization mainly introduced the filter-based backend (such as EKF) and the nonlinear optimization backend (such as graph optimization). V-SLAM tends to adopt a nonlinear optimization method with better effect and stability.

### 3.3. Loop Detection

Loop detection is used to correct errors. It can add constraints to other frames except for adjacent frames and closely relates to positioning and mapping. The backend estimates the maximum error in optimizing the data provided by the frontend, and loop detection can eliminate the influence caused by error accumulation.

**Figure 14:**
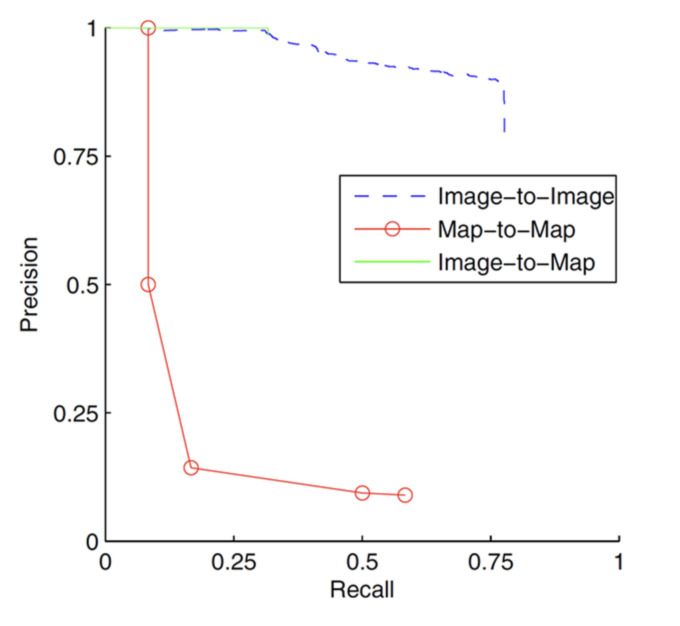
Performance comparison of three closed-loop detection methods.

Paper [57] compared three methods of loop detection for the monocular camera to complete V-SLAM and introduced three matching methods: image-to-image [58,59], map-to-map [60], and image-to-map [61]. The map-to-map matching method finds the correspondence between the identities in two submaps. The matching result indicates that although common identities between the two images can be found, the number of common features is not enough. A similar feature point needs to be found between the latest frame and the map in image-to-map matching. Among them, the research method of Cummins [62] was used to judge the position of the camera and the position of other objects through a three-point position calculation. Image-to-image matching determines a consistent point between the latest and previous images using the method of Cummins [62], which uses the graphic features obtained from the standard string vocabulary to detect the same position between two strings. The recall curve in Figure 14 shows the influence of the probability threshold on system reliability. The image-to-image method had the highest accuracy.

**Figure 15:**
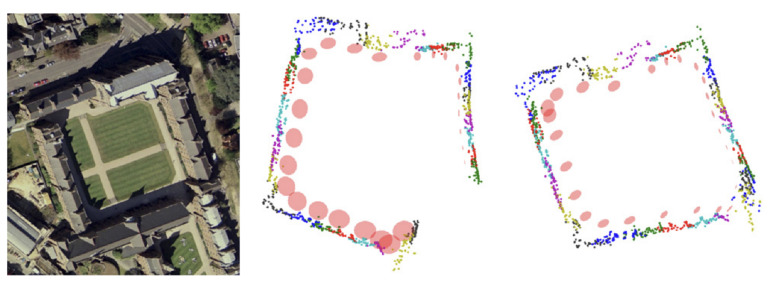
Aerial view of the courtyard and comparison before and after loop detection.

Figure 15 shows an aerial view of the Keble courtyard. By comparing the mapping before and after loop detection, the correction effect of the loop detection on the mapping is noticeable.

**Figure 16:**
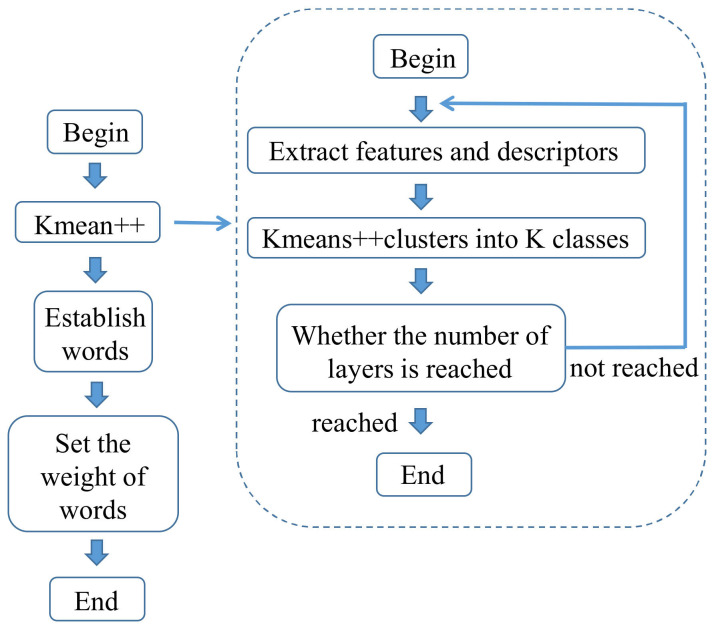
Construction steps of a visual bag of words.

In loop detection, “bag of words” (BoW) has widespread application [63,64,65,66]. Bag of words [67] refers to a technology that can use a visual vocabulary tree to turn the content of a picture into a digital vector for transmission. The steps of constructing a visual vocabulary tree [68] are shown in Figure 16.

For the newly entered image frame, each feature is traversed down from the root node of the word tree, and the node with the smallest Hamming distance is then traversed down to the leaf node. The number on each leaf node is calculated, and the image expression vector *v* is formed. The data unit structure of vector *v* is (index, value), namely (word index, weight). The weight is defined by Equation (6).

(6) $$w_{t}^{i}=tf(i,I_{t})\times idf(i),w_{t}^{i}=w_{t}^{i}/\sum w_{t}^{i}$$

where *tf*(*I*, *I_t_*) is the weight component generated when the BoW vector is generated; *idf*(*i*) is the weight component generated when the dictionary is developed.

**Figure 17:**
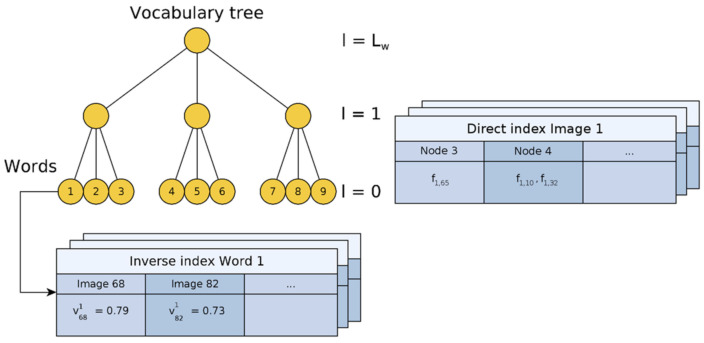
Vocabulary tree of image database.

Each word node stores a reverse index, making finding the most relevant image of the word more accessible, as shown in Figure 17. It is divided into layer *Lw*, and layer 0 represents the node where the word is located, which may contain multiple features. The inverse text frequency of node 1 is 0.79 in Image 68 and 0.73 in Image 82 in Figure 17.

After the BoW vector of the two pictures is obtained, the similarity between them is compared:

(7) $$L_{1}-scores(v_{1},v_{2})=1-\frac{1}{2}\left|\frac{v_{1}}{\left|v_{1}\right|}-\frac{v_{2}}{\left|v_{2}\right|}\right|$$

Using the database to simplify the retrieval process, the normalized score function is obtained as follows:

(8) $$\eta (v_{t},v_{t_{j}})=\frac{s(v_{t},v_{t_{j}})}{s(v_{t},v_{t-\Delta t})}$$

The candidate images that satisfy the requirements are reserved and entered into group matching for verification. The function of group matching is to prevent competition between continuous shots in the database query. After a time of consistency verification, only one group was reserved for structural consistency verification.

Data sets of New College [69], Bicocca 2009-02-25b [70], and Ford Campus [71] were used for training; Malaga6L [72] and CityCentre [62] were used for testing. Compared with FAB-MAP 2.0 [73] (the input is the disjoint image sequence, as shown in Table 2b, the test results are shown in Table 2a). By default, the algorithm achieved high accuracy, and there were no false positives in with test dataset. In the Malaga6L dataset, there was a high recall rate despite the influence of lighting and view depth. In CityCentre, the input was continuous image information. Still, the variation between the loop closed images was more significant than in the other datasets, so the recall rate was relatively low, but the accuracy rate was still 100%. See Table 2 for specific data.

Since the cumulative error always exists, loop detection is significant to the V-SLAM system. The most widely used and most effective method is the bag-of-words method, which can effectively improve and optimize accuracy and is very helpful for constructing a globally consistent map.

### 3.4. Mapping

**Figure 18:**
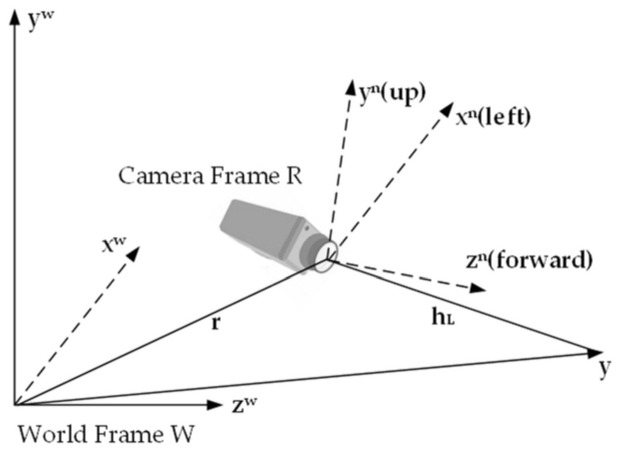
Relative position of the camera and coordinate system.

Mapping is for more precise positioning, navigation, obstacle avoidance, reconstruction, and interaction. Whether it is frontend or backend optimization, optimization makes adequate preparations for mapping. As early as 2007, Davison [15] proposed the first real-time monocular visual system with EKF as the backend, named Mono-SLAM. The most significant advantage is real-time images and no drifts. The camera and coordinate frame are shown in Figure 18.

**Figure 19:**
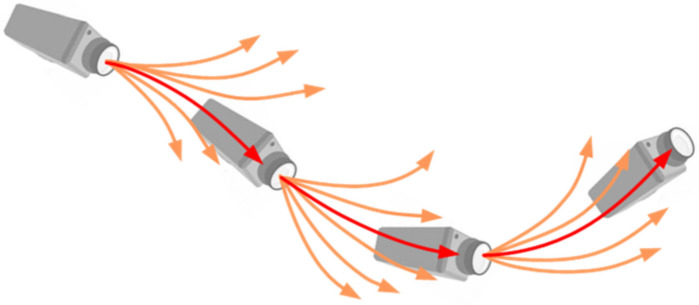
Visualization of “smooth” motion model.

**Figure 20:**
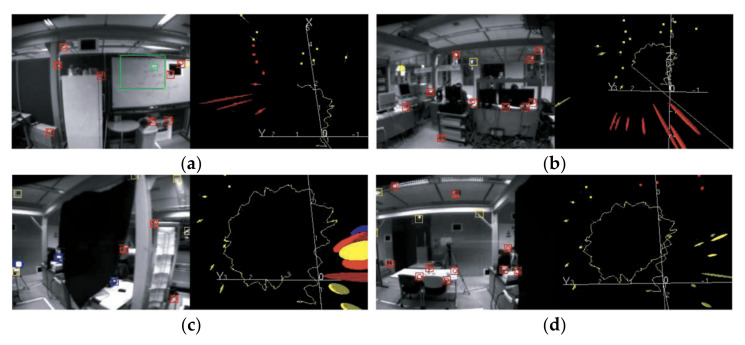
A humanoid robot walks in a circular trajectory of radius 0.75 m: (**a**) early exploration and first turn, (**b**) mapping back significantly more uncertainty; (**c**) just before loop close, maximum uncertainty; and (**d**) end of a circle with closed-loop and drift corrected.

Considering that the monocular camera cannot obtain depth information, a small amount of prior scene information is used to help the system start. A constant velocity and angular velocity model were used, as shown in Figure 19. The robot walked along a circular trajectory (yellow) with a radius of 0.75 m, as shown in Figure 20. The uncertainty increased before the loops were closed, and a drift correction was made. The disadvantage of this method is that the scene is narrow, the number of landmarks is limited, and the sparse feature points are easy to lose.

**Figure 21:**
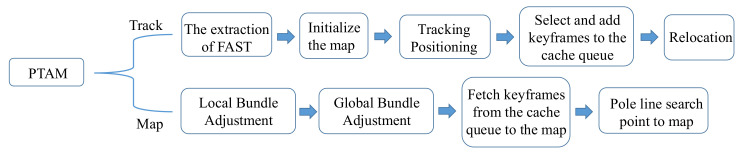
PTAM processing diagram.

**Figure 22:**
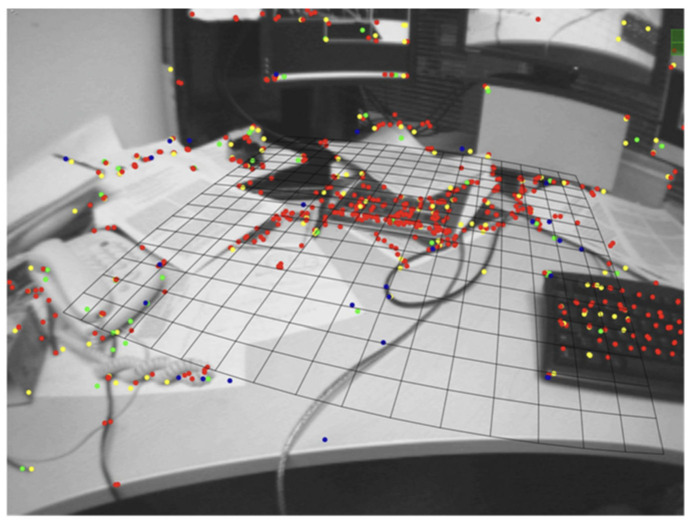
PTAM effect diagram.

Subsequently, Klein [16] proposed PTAM (Parallel Tracking Mapping) in 2007, the first V-SLAM system to process tracking and mapping in two parallel threads. It was also the first to use a nonlinear optimization backend solution, laying the foundation for the backend processing of V-SLAM to be dominated by nonlinear optimization (the process is shown in Figure 21). PTAM also proposed the Key-Frames mechanism; instead of carefully processing each image, several key images are strung together to optimize the trajectory and map. PTAM can place virtual objects on a virtual plane, and it contributed to combining AR (Augmented Reality) with SLAM. Figure 22 shows the effect of PTAM on the desktop.

**Figure 23:**
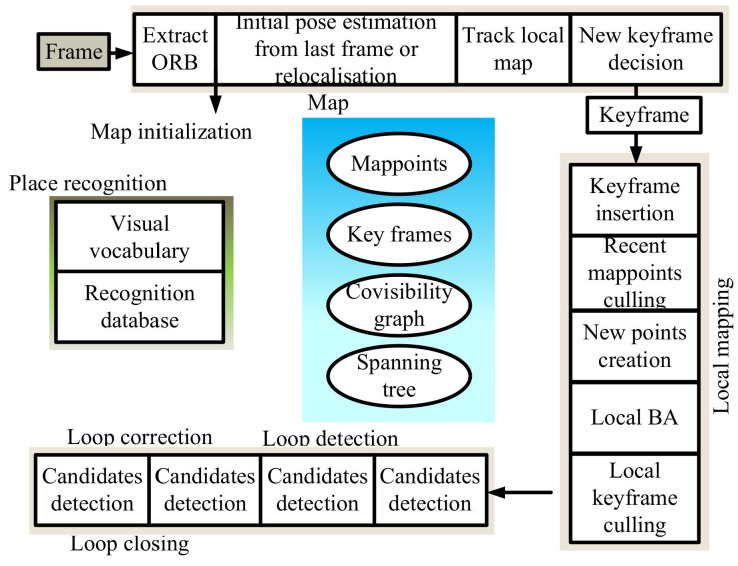
Overview of the ORB-SLAM system.

Following the parallel-thread structure [74] of PTAM, Mur-Artal [18] proposed the ORB-SLAM three-thread structure (Figure 23). It can realize the construction of sparse maps but can only satisfy the positioning demand; it cannot provide navigation, obstacle avoidance, or other functions.

**Figure 24:**
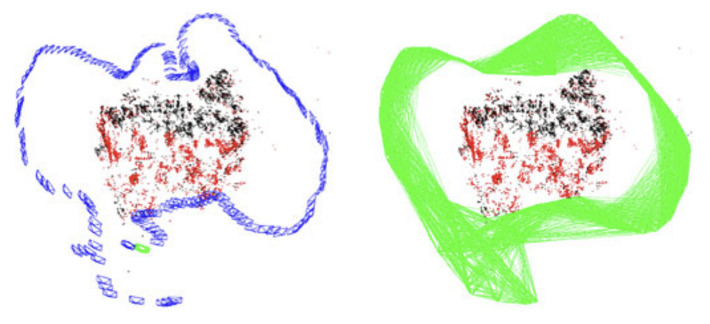
Keyframes and visibility graph of ORB-SLAM2.

The visual sensor used in ORB-SLAM [18] in 2015 was a monocular camera, which had the problem of scale drift. Based on the shortcomings of ORB-SLAM, ORB-SLAM2 [75] was proposed in 2016 as the first SLAM system for monocular, stereo, and RGB-D cameras. A thread was set up not to affect loop detection to execute a global BA [17] (Bundle Adjustment). It contained a lightweight positioning model that used VO to track the unmapped areas and match map points to achieve zero-drift positioning. Figure 24 shows ORB-SLAM2 keyframes and the visibility graph.

**Figure 25:**
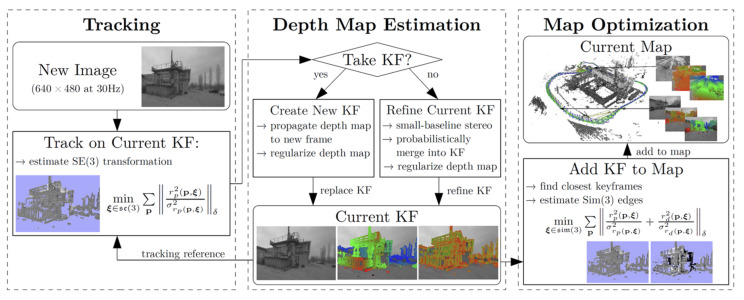
Overview of the LSD-SLAM system.

Given the long calculation time of the ORB-SLAM system, Engle [19] proposed LSD-SLAM (Large-Scale Direct Monocular SLAM) in 2014. LSD-SLAM proposes an image matching algorithm to estimate the similarity transformation between keyframes directly. It does not need to extract the feature descriptor of the image, and the transformation between two frames can be obtained by optimizing the optical measurement error. The final result is a semi-dense map, which works better in places with weaker textures. The proposal of LSD-SLAM marks the transition from sparse maps to semi-dense maps, and the process is shown in Figure 25.

**Figure 26:**
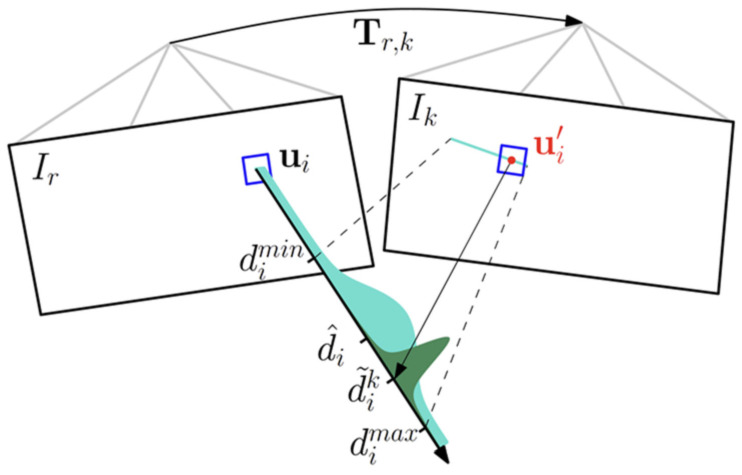
Schematic diagram of depth filter.

**Figure 27:**
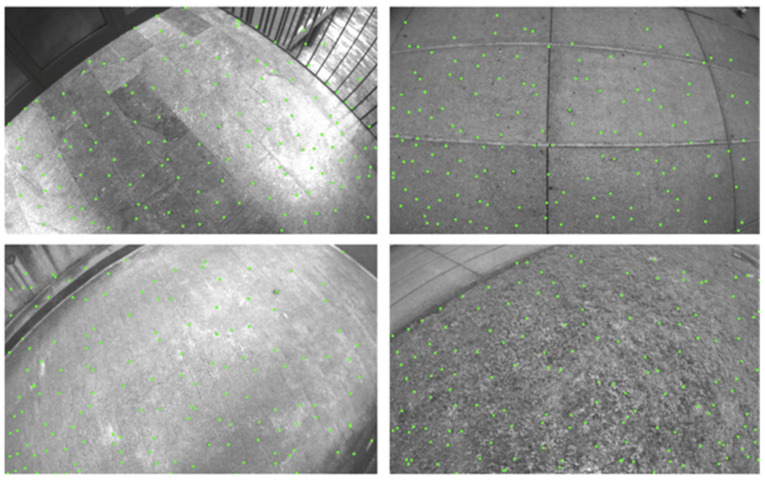
Effect diagram of SVO-SLAM.

The SVO-SLAM (Semi-direct Visual Odometry) [35] algorithm uses semi-direct visual odometry without calculating a large number of descriptors and is extremely fast. It can reach 300 frames per second on consumer laptops and 55 frames per second on a UAV (unmanned aerial vehicle). SVO-SLAM first proposed the concept of a depth filter (as shown in Figure 26) to estimate the position of critical points and use the inverse depth as a parameterized form. The schematic diagram of its effect is shown in Figure 27. The disadvantage of this method is that it discards the backend optimization and loop detection, has a cumulative error in the position estimation, and is difficult to relocate after loss.

**Figure 28:**
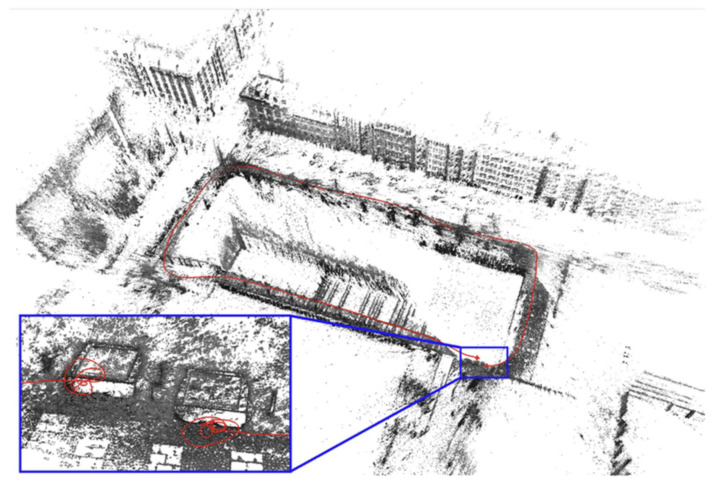
Effect diagram of DSO-SLAM.

In 2016, DSO-SLAM [20] (Direct Sparse Odometry S) was proposed and proved to be better than LSD-SLAM in terms of accuracy, stability, and speed. The algorithm did not consider prior information and could directly optimize photometric error. The optimization range was not all frames but a sliding window formed by the most recent frame and the previous few. In addition to perfecting the error model of direct method position estimation, DSO-SLAM also added an affine brightness transformation, photometric correction, and depth optimization. Still, this method did not have loop detection. Its effect diagram is shown in Figure 28. The red lines inset illustrate a cycle the start and end position, visualizing the drift accumulated during with the tracked trajectory.

The latest methods of v-SLAM have not been compared and summarized in a review article. Therefore, we extend this review to the latest methods. The results have distinguished the content of published reviews [36,37] and enriched the current state of the V-SLAM field. The latest methods are shown in Table 3, with details of the Latest algorithm, Hardware requirements, Performance, and Characteristics.

More robust performances of the novel methods have been achieved by DynaSLAM, HOOFR SLAM, PL-SLAM, DSP-SLAM, etc. The advantages of CubeSLAM are better robustness with 3D cuboid detection and edge filtering. Robust perception and the backend of DOORSLAM have been well simulated using datasets and tested field experiments. Robust 4D (3D + time) dynamic scene reconstruction has been created with DymSLAM. A robust multi-camera SLAM system (TIMA SLAM) has been developed for map accuracy. The robustness of FSD-SLAM has been better carried out with a fast movement scarce visual environment. Meanwhile, the diversification scenario has illustrated to verify its state-of-the-art novel method, such as urban areas, campuses, corridors, football stadiums, laboratories. etc.

The mapping development has gone through various V-SLAM algorithms such as Mono-SLAM, PTAM, ORB-SLAM, LSD-SLAM, SVO-SLAM, and DSO-SLAM; so, accuracy, stability, and speed in mapping have been improved. Because each has its shortcomings, choosing an appropriate mapping technology according to the application environments is necessary. Meanwhile, the latest methods have been demonstrated for more robust performances and diversification scenarios, which have enriched the current state of the V-SLAM fields.

## 4. V-SLAM Key Techniques

In the development of V-SLAM, every technical step is crucial. However, every critical technical link has technical difficulties and obstacles. Some directly affect the final positioning and mapping results of V-SLAM; so, solving the essential technologies of V-SLAM can significantly optimize the final results and provide an excellent convenience for its application.

### 4.1. Feature Detection and Matching

The primary purpose of the frontend of V-SLAM is to provide better data for the backend, and, in this, the visual odometer [25] plays a key role. It determines the position and direction of the robot by analyzing the camera image. Then, depending on whether features need to be extracted, it can be divided into a feature point method or a direct method. Feature point is most commonly used because of its high stability [63,85,86].

Photos and other kinds of original information are continuous analog signals; so, they must be transformed into a digital form for a computer to process the data. Digital images are often stored in the form of a gray matrix, but the gray value will change with the lighting factors and object materials. To further study the identification and positioning of critical points, it is necessary to select those points in the image that will not change with a change of perspective, namely, feature points. In some scenes, the feature points of the image do not meet the requirements; so, more stable human-designed feature points are generated. The most common features are SIFT [87], SURF [88], and ORB [89].

#### 4.1.1. SIFT

**Figure 29:**
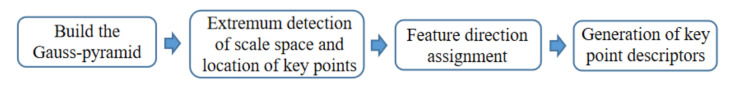
Extraction steps of SIFT features.

SIFT (Scale Invariant Feature Transform) [87] is a classic feature point with image-rotation and size invariability. It also has robustness to image noise and a visible illumination change [85,90,91,92]. However, the large dimension of feature points makes it challenging to complete a real-time and accurate calculation, much less rapid positioning and mapping. The extraction steps of SIFT features are shown in Figure 29.

**Figure 30:**
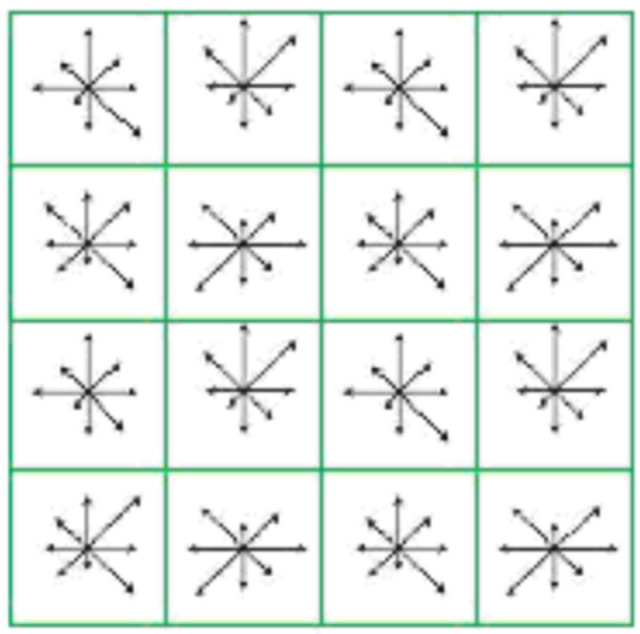
128-dimensional SIFT feature vector.

**Figure 31:**
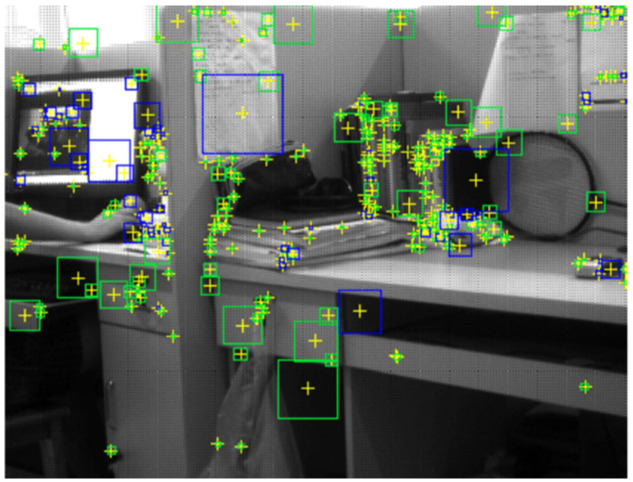
SIFT keypoint descriptors.

The 16 × 16 rectangular-block pixels around the key points are selected and divided into 4 × 4 subareas. A feature point generates SIFT feature vectors of 4 × 4 × 8 dimensions. When using the SIFT algorithm, the distance between each feature point of the first image and all feature points of the second image needs to be calculated. Feature vectors and descriptors are shown in Figure 30 and Figure 31.

#### 4.1.2. SURF

**Figure 32:**
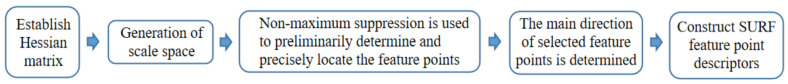
Steps of SURF feature algorithm.

SURF (Speeded Up Robust Features) [88] was proposed based on the SIFT algorithm. It was mainly improved to deal with the disadvantages of the slow operation speed and extensive computation of the SIFT algorithm. The SURF feature is widely used as a feature extraction and analysis method for V-SLAM [86,93,94], and the algorithm flow is shown in Figure 32.

Firstly, a Gaussian pyramid scale-space needs to be constructed. SURF adopts determinant approximation images of a Hessian matrix, which is defined as:

(9) $$H(f(x,y))=\left[\begin{array}{cc} \frac{\partial^{2}f}{\partial x^{2}} & \frac{\partial^{2}f}{\partial x\partial y}\\ \frac{\partial^{2}f}{\partial x\partial y} & \frac{\partial^{2}f}{\partial y^{2}} \end{array}\right]$$

**Figure 33:**
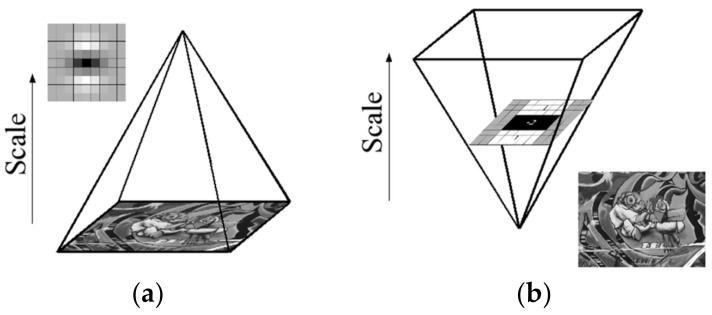
Different variations in scale space: (**a**) SIFT: filter remains unchanged, image size is changed, (**b**) SURF: image size is unchanged, and the filter size is changed.

The pyramid image has many layers, and each has images with different scales. Figure 33a is the traditional way to build a pyramid structure. It changes the image size and uses a Gaussian function to smooth the sublayers repeatedly. The SURF algorithm only changes the filter size, omits the downsampling process, and improves the processing speed (Figure 33b).

**Figure 34:**
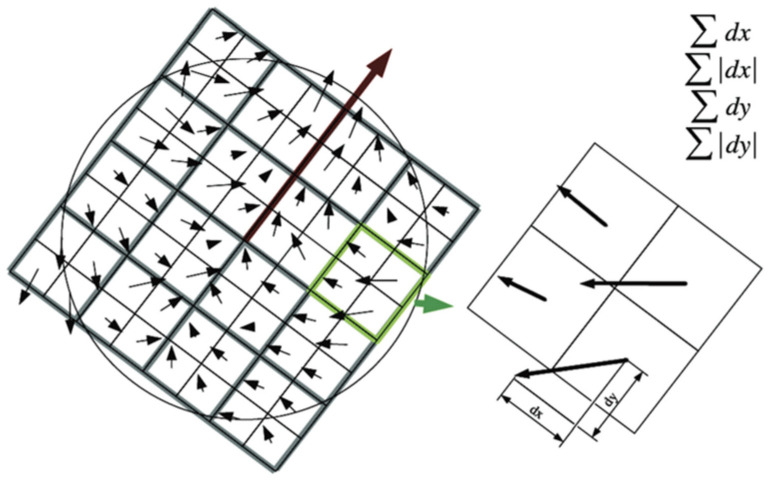
Construction of SURF feature point descriptors.

The pixel points processed by the Hessian matrix are compared with the 26 points in the three-dimensional field, and those with the most vital features are selected as feature points. A square box is taken around the feature point and divided into 16 subareas. Each subarea calculates the horizontal and vertical Haar wavelet features of 25 pixels. The process is shown in Figure 34. Each feature point is a vector of 16 × 4 = 64 dimensions, which is half the size of SIFT.

#### 4.1.3. ORB Feature

**Figure 35:**
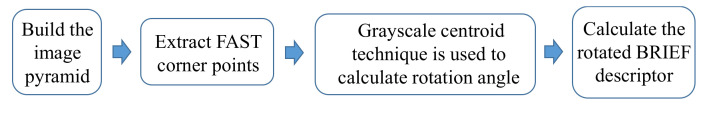
Steps of the ORB feature algorithm.

ORB (Oriented FAST and Rotated BRIEF) [89] is the combination of a FAST [95] (Features from Accelerated Segment Test) feature detection operator and a BRIEF [96] (Binary Robust Independent Elementary Features) descriptor. The FAST is a feature point with a breakneck calculation speed. It is mainly applied to detect a noticeable change in gray level in local images, but the repeatability is not strong, and the distribution is not uniform. A BRIEF is a binary string that improves the accuracy of real-time feature detection and data extraction. Many V-SLAM algorithms adopt ORB features [97,98], and the flow is shown in Figure 35.

**Figure 36:**
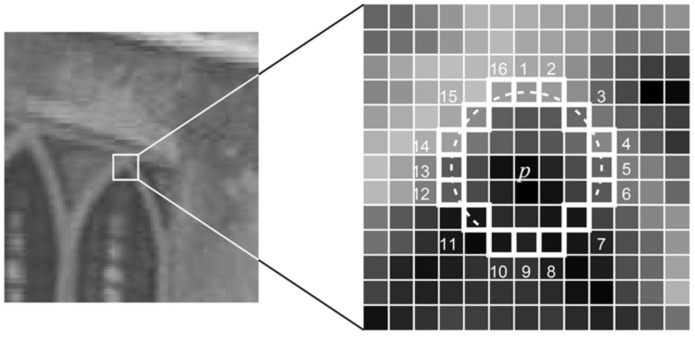
A 12-point segment test corner detection in an image patch.

To determine whether pixel point P is a crucial point of FAST, we only need to judge whether the difference between the gray value of N consecutive points and P among the 16 surrounding pixels exceeds the threshold [95]. The positions of the 16 points are shown in Figure 36. After finding the key points, the grayscale centroid technique is used to calculate the direction of the feature.

The result of the BRIEF algorithm is a binary string. To increase the noise resistance of feature descriptors, Gaussian smoothing is needed for the image. With the feature point *p* as the center, a neighborhood window of S × S is taken, and a pair of points *p_i_* and *q_i_* are randomly selected to compare the two points’ pixel sizes. If *I*(*p_i_*) > *I*(*q_i_*), 1 in the binary string will be generated; otherwise, 0. N pairs of points are randomly selected in the window, and the above steps are repeated to form a binary code. The code describes the feature points, namely, the feature descriptor.

The ORB feature point is the most commonly used because of its speed. However, appropriate feature points should be selected according to different visual information and scene requirements. Correct feature matching relieves much of the burden of backend optimization.

### 4.2. Selection of Keyframes

**Figure 37:**
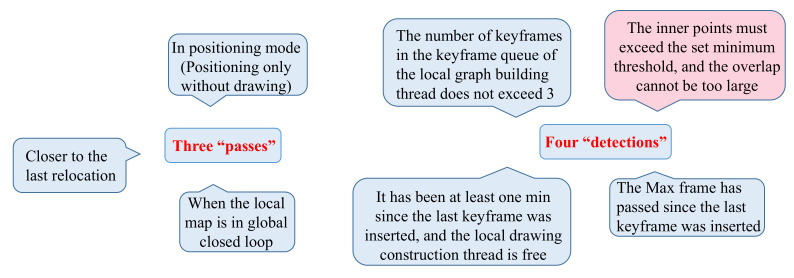
Principle of “three passes, four detections”.

If there are errors in position estimation, the frame-to-frame alignment method will cause cumulative floating. Therefore, a V-SLAM based on critical frames [99] was proposed. Keyframes play a role in filtering to prevent useless or wrong information from entering the optimization and damaging the positioning construction’s accuracy. The screening of keyframes follows the principle of “three passes, four detections” (Figure 37). Based on satisfying the requirements of the internal points, one of the other three detection conditions should be satisfied.

**Figure 38:**
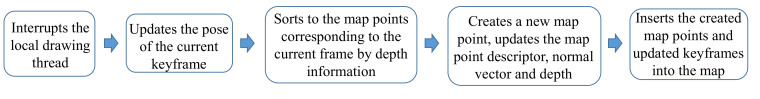
Flow diagram for inserting keyframes.

After filtering, if the current frame is a keyframe, it should be input into the local diagram building and loopback detection modules. The critical frame insertion process is shown in Figure 38.

Christian [99] proposed a keyframe selection method based on entropy similarity, which can reduce position floating. The entropy ratio α was submitted to treat mismatch in the course of a trajectory and the different scenes. It can be calculated by the ratio of parameter entropy *H*(*ξ_k,k+j_*) (the motion estimation from the previous keyframe *k* to the current frame *j*) to the parameter entropy *H*(*ξ_k,k_*_+1_) (the motion estimation from the keyframe *k* to the next frame *k* + 1), as follows:

(10) $$\alpha =\frac{H\left(\xi_{k:k+j}\right)}{H\left(\xi_{k:k+1}\right)}$$

**Figure 39:**
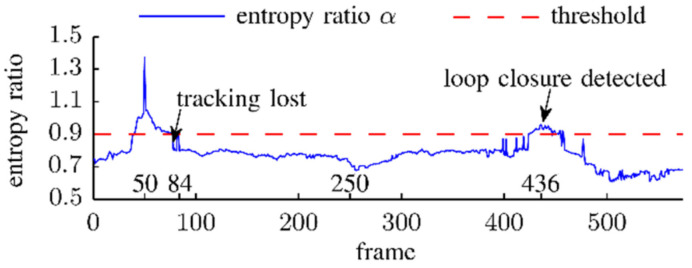
Entropy ratio α from frame 50 to other frames.

Figure 39 shows the entropy ratio from frame 50 to all other frames in the fr1/desk dataset. If *α* falls below the predefined threshold for the current frame, the previous frame is selected as the new keyframe and inserted into the map.

Keyframe selection strategy is an essential factor for algorithm performance, significantly reducing the calculation pressure of the backend optimization. The effective selection of keyframes contributes to better positioning and image building.

### 4.3. Uncertainty Technology

**Figure 40:**
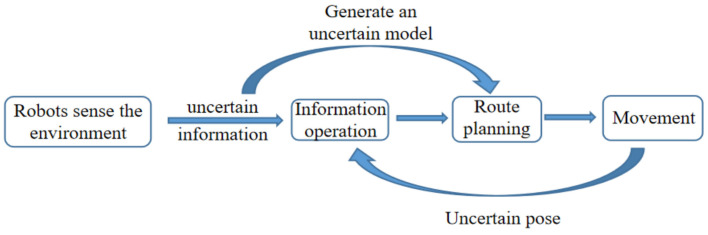
System processing uncertain information.

The uncertainty of perception information may come from an imperfection, such as blocking a reference object. It may also come from uncertainty brought about by randomness—for example, the influence of unknown external forces such as mobile robot roller slippage, sensor parameters (such as resolution), or observation noise. Since the mobile robot must rely on sensors to obtain information when the environment is unknown, the uncertainty of perceptual details will lead to inaccuracy in the constructed environment model, as shown in Figure 40.

**Figure 41:**
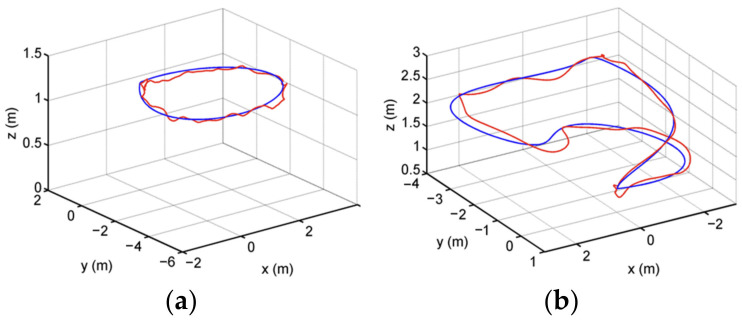
Trajectory tracking in the flying arena during trajectory tracking: (**a**) circular trajectory; (**b**) 3D trajectory.

The measurement error of the sensor is related to the measurement accuracy and the conditions and times of measurement. As for the uncertainty of sensor observation data, high resolution or multisensor data fusion can be adopted to solve the problem and minimize error accumulation. For example, in paper [8], visual and inertial sensors were combined to comprehensively process images and measurement information to reduce errors caused by objective uncertainties. Visual Inertial Odometry (VIO) integrates a Visual Inertial Measurement Unit (IMU) and VO. Paper [8] described a vision-based localization and environment mapping and MPC (Model Predictive Control) trajectory tracking with obstacle avoidance for autonomous MAV navigation in GPS-denied cluttered environments. The flight-tested micro aerial vehicle performed automatic takeoff and landing and autonomous obstacle avoidance trajectory tracking. The tracking trajectory on the flight field is shown in Figure 41 (blue: reference trajectory, red: MAV (micro air vehicle trajectory). The positioning and tracking accuracy during the flight is shown in Table 4, and the data show that the positioning and tracking accuracy was very high, with an error as low as 6 cm.

Uncertainty comes from various sources, which affects the final mapping. Different sensors have different characteristics and advantages, so adopting multisensor fusion can reduce the negative impact. Meanwhile, some new algorithms are able to minimize the influence of uncertainty on V-SLAM.

### 4.4. Expression of Maps

Mobile robots sense the surrounding environment through sensors and eventually build environmental maps. Since map building mainly serves for positioning, it is necessary to create maps according to different environmental requirements. The construction methods of an environment map mainly include a grid map, a topological map, and an octree.

**Figure 42:**
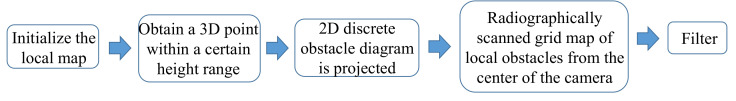
Establishment process of a 2D grid map.

A two-dimensional grid map [100] is widely used in the navigation field of mobile robots, such as path planning and real-time obstacle avoidance. An RGB-D camera can obtain a 3D point cloud of the scene in real-time and establish a local grid map using depth information, as shown in Figure 42.

**Figure 43:**
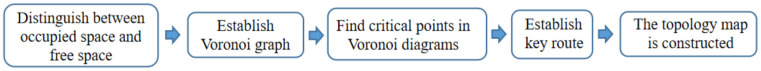
Establishment process of a topology map.

A topology map is based on a raster map, abstracting the environment into a graph model [101]. A grid-based map is a set of small areas separated by narrow passages, such as doorways, known as critical boundaries. The segmented submap is mapped to the same-type map, where nodes corresponding to regions and arcs connect adjacent areas. The establishment steps are shown in Figure 43.

**Figure 44:**
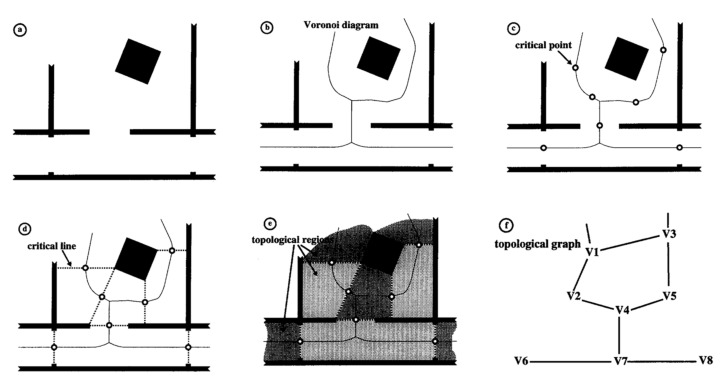
Extraction of a topology map: (**a**) Metric map; (**b**) Voronoi diagram; (**c**) critical points; (**d**) Critical lines; (**e**) Topological regions, and (**f**) Topological graph.

Figure 44a is a metric map where cells with occupancy values below the threshold in the occupancy grid are considered free space (denoted by C). For each point (*x*,*y*) in C, there are two nearest points in C, named the base point of (*x*,*y*). The distance between (*x*,*y*) and its base point is the gap of (*x*,*y*). Figure 44b below depicts a Voronoi map [102]. The key to dividing free space is to find critical points, and Figure 44c represents the vital points. The required boundary is obtained by connecting each crucial point with its reference point (Figure 44d). Figure 44f is an example of a topology map.

**Figure 45:**
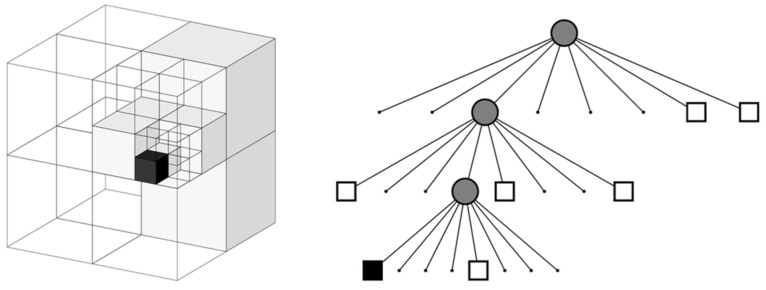
Diagram of octree.

**Figure 46:**
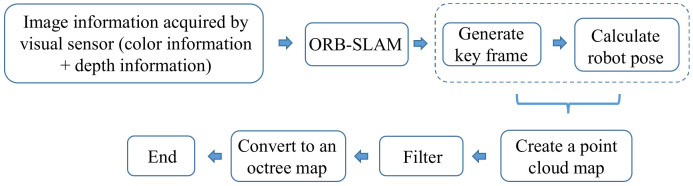
Establishment process of octree map.

An Octo Map [103] is a flexible, compressed, and constantly updated map. Each octree node represents a space in a cubic volume, called a voxel. The block is recursively subdivided into eight subblocks until a given minimum voxel size is reached, as shown in Figure 45. The minimum voxel size determines the resolution of the octree. The mapping construction process is shown in Figure 46.

**Figure 47:**
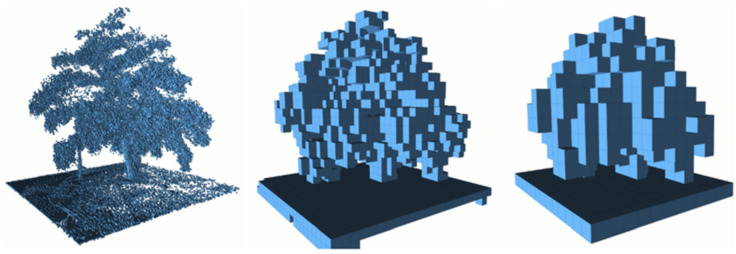
Display effect of 0.08, 0.64, and 1.28 m resolutions.

Figure 47 shows an example of querying an octree map of active voxels at several different resolutions, where multiple solutions of the same map can be obtained at any one time by limiting the depth of the query.

Environmental map types mainly include grid, topology, and octree. V-SLAM should also selectively determine the kind of final map based on different scene types and sensor types.

## 5. Developmental Needs for V-SLAM

V-SLAM has been developing towards stronger robustness and real-time performance in recent years. More and more new technologies have emerged making V-SLAM more widely used. However, it still faces many significant challenges in a diversified application environment.

When visual sensors acquire indoor and outdoor information, similar scenes are inevitable. Environmental factors lead to low accuracy in feature matching; so, it is necessary to improve the quality of the original image acquisition and reduce the influence of the external environment.

The key to the accuracy and efficiency of global map construction is the selection of high-precision keyframes, which determine the amount of backend optimization tasks and the graphics construction effect of V-SLAM. Therefore, accuracy and efficiency in handling valid keyframes are required.

Due to the uncertainty caused by a single sensor, multisensor fusion is needed to solve the problem. At the same time, improved data coupling and use are required. A new map model based on the existing map form needs to be developed for a multitasking and complex scene.

With the development of new V-SLAM systems, new multisensor fusion, and new algorithms, it is believed that V-SLAM will play an essential role in “unmanned operations” and continue to explore new developmental directions.

## 6. Conclusions

V-SLAM has become a hot spot for solving localization and mapping in autonomous navigation without human intervention. It is necessary to classify and summarize the research status of V-SLAM for more in-depth explorations.

The V-SLAM originated from SLAM with visual sensors. Therefore, the historical milestones of SLAM and V-SLAM were outlined.

Five parts of the classic V-SLAM framework were explained separately, including frontend (visual sensor and visual odometry), backend optimization, loop detection, and mapping. Each section detailed the current state of the V-SLAM fields. Meanwhile, the details of the latest methods are shown; the novel VI-SLAM methods were reviewed and extended.

The four critical techniques of V-SLAM and their technical difficulties were summarized in context, including feature detection and matching, the selection of keyframes, uncertainty technology, and expression of maps. The key results were extracted from the literature.

Finally, the development direction and needs of V-SLAM research were proposed for higher accuracy positioning, a lower runtime, and stronger robustness.

## Figures and Tables

**Table 1:** Comparison of parameter changes before and after correction.

	*σ*	*ρ*
before correction	0.049	0.033
after correction	0.046	0.032

**Table 2:** Comparison of accuracy and recall rate: (**a**) accuracy and recall rate of test system; (**b**) accuracy and recall rate of FAB-MAP2.0.

(a)				(b)				
Precision and Recall of Our System				Precision and Recall of FAB-MAP 2.0				
Dataset	# Images	Precision (%)	Recall (%)	Dataset	# Images	Min. p	Precision (%)	Recall (%)
NewCollege	5266	100	55.92	Malaga6L	462	98%	100	68.52
Bicocca25b	4924	100	81.2	CityCentre	2474	98%	100	38.77
Ford2	1182	100	79.45					
Malaga6L	869	100	74.75					
CityCentre	2474	100	30.61					

**Table 3:** Demonstration latest V-SLAM methods.

Latest Algorithm	Hardware Requirements	Scenario	Performance	Characteristics
DynaSLAM [76]	monocular, stereo and RGB-D	dynamic scenarios; static map	tracked trajectory: >87.37%; average of the RPE: 0.45%	Dynamic object detection; Background inpainting; Multi-view geometry, deep learning.
HOOFR SLAM [77]	multi and stereo camera;	urban; Karlsruhe; campus	CPU time: 62.235 ms; GPU time: 36.154 ms	Large-scale unknown environment; Hardware-software mapping; Low-power.
PL-SLAM [78]	stereo camera;	rooms, industrial scenario	Runtime: 57.05 ms (KITTI); Runtime: 37.48 ms (EuRoC)	Points and line segments; More diverse in 3D elements; Lower computational time.
CubeSLAM [79]	monocular camera	Indoor; outdoor	Mean tans error: 4.42 m; Mean depth error: 4.9%; Runtime: 365.2 ms	3D cuboid object detection; Long-range geometric and scale; Static and dynamic scenes.
DOORSLAM [80]	two quadcopters featuring stereo cameras	football field;	Threshold (1%): ATE (2.1930 m); Threshold (75%): ATE (18.255 m)	Peer-to-peer communication; Distributed pose graph optimization; Outlier rejection mechanism.
DymSLAM [81]	stereo camera;laser scanner;	indoor; corridors	RSEM of moving object: Position [cm]: 10.81; Rotation [°]: 2.0472	4D (3D + time) dynamic scene; Improving accuracy of boundary; Dynamic environment.
TIMA SLAM [82]	multi-camera System	hall; laboratory; corridors	EuRoC/ASL: 0.023; KITTI odometry: 0.58	Independent tracking; Without precalibration; Better compatibility.
FSD-SLAM [83]	monocular camera	indoor	ATE: 0.018793 m RPE: 0.028753 m	Accurate camera pose estimation; Enhanced dynamic covariance scaling; Point cloud integration.
DSP-SLAM [84]	monocular, stereo, stereo + LiDAR	cars; chairs	Faster iteration time: 4 s; Fewer iterations: 10	Pose and shape with less drift; Maintaining a consistent global map; Almost real-time performance.

**Table 4:** Localization and tracking accuracy in a flying arena.

	RMS of Localization Error (m)	RMS of Tracking Error (m)
Circular trajectory	0.067	0.131
3D trajectory	0.077	0.219

## Data Availability

Not applicable.
